# Investigating the Degradation of Historical Man‐Made Cellulose‐Derived Textiles via Accelerated Ageing

**DOI:** 10.1002/cplu.202500025

**Published:** 2025-05-07

**Authors:** Louise Garner, Simoní Da Ros, Katherine Curran

**Affiliations:** ^1^ UCL Institute for Sustainable Heritage University College London 14 Upper Woburn Place London WC1H 0NN UK; ^2^ WMG The University of Warwick Gibbet Hill Road Coventry CV4 7AL UK

**Keywords:** carbohydrates, cellulose acetate, infrared spectroscopy, man‐made cellulose textiles, regenerated cellulose

## Abstract

Cellulose‐derived materials, like paper and cellulose acetate, are known to be vulnerable to degradation within museum collections. Studies have been conducted and degradation markers have been identified on these materials. However, the degradation of man‐made cellulose‐derived fibers in collections is not well understood. This study aims to provide insights into historical cellulose acetate and regenerated cellulose textiles to quantify their physical and chemical changes during degradation using accelerated ageing experiments. Potential physical and chemical markers for degradation are identified, including changes in surface morphology, mass loss, discoloration and changes in spectral bands. These markers can be used to improve understanding of the degradation mechanisms of historical cellulose acetate and regenerated cellulose textiles and guide the development of conservation strategies. These findings have important implications for understanding the stability of man‐made cellulosic fibers in museum collections.

## Graphical abstract

Cellulose‐derived materials, such as paper and cellulose acetate, degrade in museum collections. The degradation of man‐made cellulose fibers remains poorly understood. This study examines historical cellulose acetate and regenerated cellulose textiles using accelerated ageing. Identified markers, for instance surface changes, mass loss, discoloration, and spectral shifts, provide enhanced understanding of degradation behavior and inform conservation and preservation strategies.

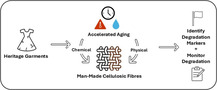

## 1 Introduction

Ensuring the long‐term preservation of heritage objects requires a thorough understanding of material degradation processes, including the investigation of underlying mechanisms and their associated degradation markers. Currently, there is a well‐established appreciation of the complexities involved in the preservation of modern materials.^[^ ^1^, ^2^, ^3^ ^]^ Modern materials, such as plastics, films, and synthetic or semisynthetic textiles, pose distinct preservation challenges due to their complex formulations and interactions with environmental factors.

While significant research has been devoted to solid plastics and films, the stability of cellulose‐derived semisynthetic textiles, such as viscose rayon and cellulose acetate fibers, remains under‐explored.^[^ ^3^, ^4^, ^5^, ^6^, ^7^ ^]^ The manufacturing of regenerated cellulose fibers (RCFs) began in 1892, marking one of the earliest developments in man‐made fibers. Viscose rayon, the oldest form of RCFs, initially dominated the textile industry but was gradually surpassed by fully synthetic fibers in the mid‐20th century.^[^ ^8^ ^]^ RCFs were widely used in faux fur, velvet, taffetas, and linings, spanning applications from everyday garments to haute couture.^[^ ^1^ ^]^ As a result, a significant number of 20th‐century textiles in museum collections contain these fibers. Equally, as the textile industry shifts toward more sustainable and biodegradable options, museums are likely to encounter increasing numbers of modern RCFs in their collections.^[^ ^8^ ^]^

Cellulose acetate fibers (CAFs) are another prominent man‐made cellulosic fiber (MMCF) found in museum collections, commonly in the form of photographic films and three‐dimensional plastic objects.^[^ ^1^ ^]^ Adopted as a textile in the 1920s, CAFs are frequently found in historical garments. Like their use in films and plastics, CAFs exist in two forms: cellulose diacetate and cellulose triacetate. Both forms have been widely utilized across various applications, yet CAFs differ chemically from other cellulose‐based materials. The cellulose structure in CAFs is modified by substituting hydroxyl groups with acetyl groups during production.^[^ ^9^ ^]^ While the degradation of cellulose acetate films and plastics has been thoroughly studied, including the identification of degradation markers and development of ageing models, there is limited research that translates these findings to cellulose acetate textiles, which warrants further investigation.

**Figure 1:**
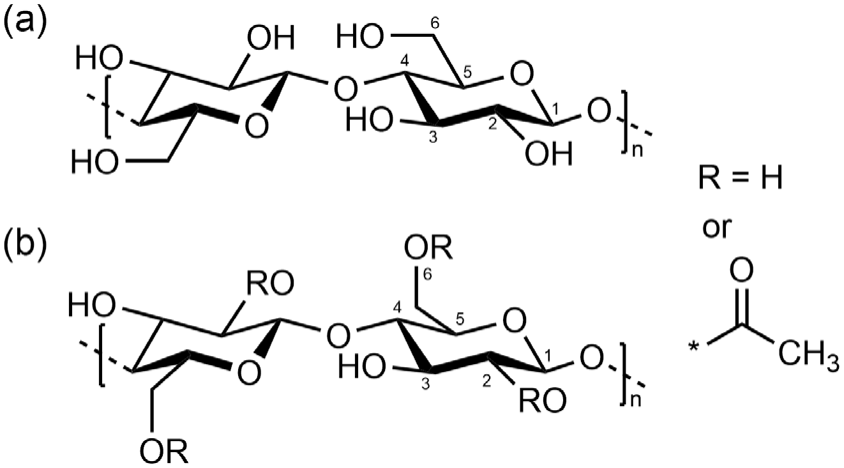
a) Chemical structure of cellulose, b) chemical structure of cellulose acetate.

RCFs and CAFs are both cellulose‐derived materials (**Figure** **1**), but they differ in their manufacturing processes and resulting properties. For RCFs, the first stage, known as mercerization, consists of the swelling and dissolution of cellulose pulp in a solution of NaOH, which is thought to form hydrates with the water molecules and supports the breaking of hydrogen bonds.^[^ ^10^ ^]^ At this point, an intermediate of alkali cellulose is formed, and changes in the supra‐molecular structure, such as crystallinity and degree of polymerization (DP), properties occur. The alkali cellulose undergoes the xanthation process, where the pulp is exposed to CS_2_ to produce sodium cellulose xanthate.^[^ ^11^ ^]^ The cellulose xanthate is further dissolved in NaOH to form the viscose pulp. Subsequently, the viscose is filtered, spun, and dried to form fibers. This process yields a fiber with a higher amorphous content and a lower DP compared to natural cellulose fibers like cotton.^[^ ^1^, ^9^ ^]^ The increased amorphous content of RCFs contributes to higher susceptibility to moisture due to the hydroxyl groups and high proportion of amorphous material, which may influence their degradation over time. Further research is needed to clarify the specific degradation patterns that result from these properties.

In contrast, CAFs are manufactured by chemically modifying cellulose through acetylation. This process involves replacing some of the hydroxyl groups in the cellulose structure with acetyl groups by treating the cellulose with acetic anhydride.^[^ ^11^ ^]^ Depending on the degree of acetylation, the fibers can be classified as either cellulose diacetate or cellulose triacetate, each with different properties. This can be described as the degree of substitution (DS) which indicates the average number of hydroxyl groups replaced by acetyl groups during cellulose acetylation. The acetylation process results in a more hydrophobic fiber than RCFs, with a higher resistance to moisture.^[^ ^1^ ^]^ Despite extensive studies on cellulose acetate in the context of films and plastics, relatively little is known about how these properties affect the long‐term stability of CAFs within museum collections.

However, the conservation community has long recognized the inherent instability of other cellulose‐derived materials within heritage collections. For example, cellulose acetate (CA) and cellulose nitrate (CN) plastics are known to experience degradation processes such as deacetylation and hydrolysis, releasing acetic or nitric acids that accelerate their deterioration and create storage challenges.^[^ ^2^, ^3^, ^6^, ^12^ ^]^ Similarly, paper and cotton canvases—native cellulose materials—have exhibited vulnerability to environmental factors such as acidity, moisture, and light, resulting in loss of mechanical integrity, discoloration, and chemical instability over time.^[^ ^13^, ^14^, ^15^ ^]^ These studies emphasize the necessity of conducting similar research on cellulose‐derived fibers to enhance our understanding of their degradation pathways.

Furthermore, cellulose‐derived materials exhibit heightened susceptibility to degradation due to interactions with additives. For instance, CA is particularly prone to plasticizer loss, a process that accelerates its breakdown and compromises its mechanical integrity.^[^ ^16^ ^]^ Likewise, the degradation of paper is significantly exacerbated by the presence of certain inks, such as iron gall ink, which initiates oxidative and acidic reactions, resulting in accelerated chemical and physical deterioration.^[^ ^17^, ^18^ ^]^ These examples demonstrate the complex relationship between cellulose‐based materials and additives, emphasizing the need for a deeper understanding of various types of cellulose materials. Addressing this knowledge gap not only supports the broader preservation of cellulose‐derived materials but also improves our ability to mitigate risks associated with material deterioration in cellulose‐derived garments within heritage collections.

The heterogeneity of modern textile garments also presents a significant challenge for preservation. Blends can be achieved through the combination of yarn spun from multiple fiber types or through the weaving of various fiber types into a single fabric.^[^ ^19^ ^]^ The textile industry has consistently used blends to tailor the properties of fabrics.^[^ ^20^ ^]^ A common example of this is the addition of elastane to add stretch to fabric. Textile and fiber blends present a considerable challenge not only with identification but also contribute to the complexity of the degradation of man‐made textiles.^[^ ^20^, ^21^ ^]^ Previous studies have documented the presence of fiber blends and the combination of multiple materials within a single garment, complicating the assessment of degradation processes.^[^ ^22^ ^]^ The degradation of such complex objects raises important questions regarding their long‐term stability, storage requirements, and appropriate treatment strategies.

**Figure 2:**
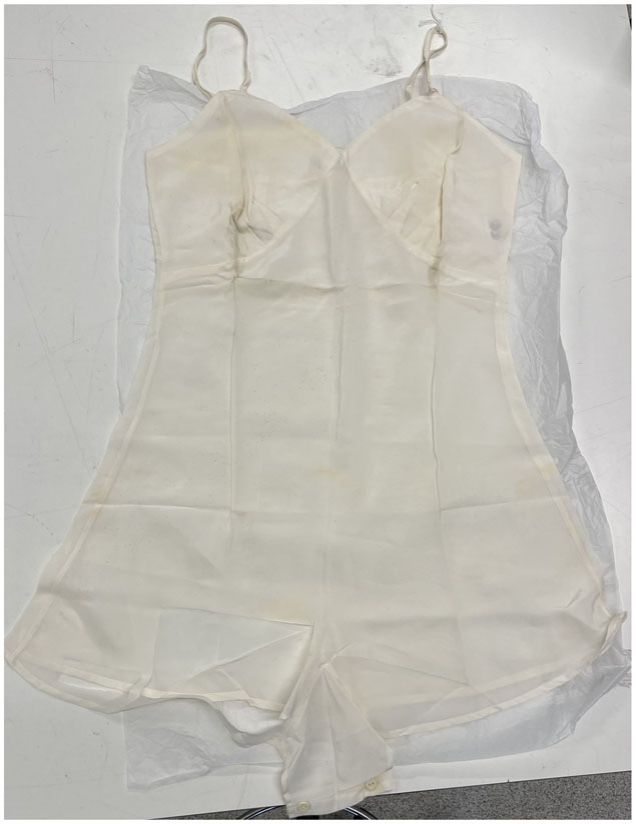
Example of garment samples (CAF‐RCF), see all garment images in Figure S1, Supporting Information.

In this study, we aim to address these concerns by investigating the degradation of historic RCFs and CAFs, both in isolation and as blends. As two of the earliest man‐made fibers, these materials represent some of the oldest semisynthetic garments encountered in collections and are potentially among the most vulnerable textiles due to their age. Our objective is to provide a clearer understanding of their physical and chemical degradation markers building on existing techniques applied to cellulose‐derived materials to characterize the degradation of heritage objects. Using samples from garments dating to the 1940s and 1950s (**Figure** **2**), the findings of this study can be situated in relation to analogous materials that have been more extensively researched, such as solid cellulose acetate plastics and native cellulose present in materials like paper, cotton, and wood.

By conducting accelerated ageing experiments on deaccessioned museum objects, we aim to simulate the degradation mechanisms frequently encountered by museum garments and assess the potential impacts on the preservation of similar textiles in the future. Accelerated ageing employs harsh conditions to expedite degradation, enabling the observation of degradation markers and behavior in a measurable timeframe. Accelerated ageing has been extensively utilized in studies of both polymeric and cellulose heritage to assess and quantify degradation.^[^ ^15^, ^16^, ^23^, ^24^, ^25^, ^26^, ^27^ ^]^ The study utilizes accelerated ageing through elevated temperatures (70 °C) and high relative humidity (78% RH) for 120 days. Throughout the experiment, we monitored both physical and chemical changes in the samples. Our goal is to contribute to the improved understanding of the degradation trends of RCFs and CAFs and their blends.

## 2 Results and Discussion

### 2.1 Characterization of Garment Condition

Prior to artificial ageing, all garments underwent comprehensive characterization to understand their initial condition. Five garments were selected for analysis, comprising two CAF garments (CAF‐1, CAF‐2), two RCFs garments (RCF‐1, RCF‐2), and one CAF‐RCF blend (**Table** **1**) (for full images of the garments see SI 1 in the supplemental information). For comparative purposes, an additional new RCF sample (RCF‐3) was included to assess the effects of artificial ageing alongside the naturally aged RCF samples. However, due to their reduced use in the current textile industry, new CAFs or CAF‐RCF blend samples were not available for inclusion in this study. The garments, dated based on their labels to ≈70–80 years of age, were generally well preserved, though localized degradation, primarily discoloration, was observed. Samples for analysis were taken from regions of the garments that showed no visible signs of degradation. The initial DS of cellulose acetate garments, measured at the beginning of the study using attenuated total reflectance Fourier transform infrared (ATR‐FTIR) spectroscopy, was found to be 2.42. However, this measurement does not definitively establish the original DS or determine if the garments are cellulose diacetate or triacetate, as natural ageing may have led to deacetylation that altered the original substitution level.

**Table 1:** Sample information, including fiber type, weave structure, and textile color. Each sample represents three repeats from each garment.

Sample	Fiber Type	Weave structure	Color
RCF‐1	Regenerated cellulose	Plain	Pink/White
RCF‐2	Regenerated cellulose	Plain	White
RCF‐3a)	Regenerated cellulose	Plain	White
CAF‐1	Cellulose acetate	Satin	Pink
CAF‐2	Cellulose acetate	Satin	White
CAF‐RCF	Cellulose acetate‐regenerated Cellulose blend	Plain	White

a) RCF‐3 is an unaged reference for regenerated cellulose fibers, all other samples are from 1940‐50.

**Figure 3:**
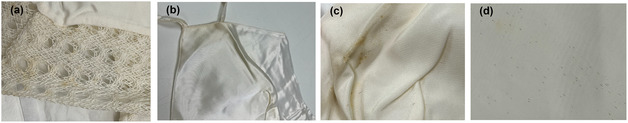
a) RCF‐1 showing discoloration of cotton lace, b) CAF‐1 showing yellowing of edge of slip dress component, c) CAF‐RCF showing browning and yellowing around fold of slip dress, and d) CAF‐RCF showing spotting across main fabric.

All garments were found to be in generally good condition, with no visible signs of mechanical damage. All samples containing cellulose acetate, including the blend, exhibited a strong acetic acid odor. The primary indication of ageing was localized discoloration across several regions of the garments. Notably, CAF‐1 and RCF‐2 exhibited less pronounced discoloration, which may partly be attributed to their original pink color, potentially complicating the assessment of their condition. In the case of RCF‐2, mild discoloration was observed, though this was primarily confined to browning of the decorative cotton lace rather than the RCFs fibers themselves (**Figure** **3**a). CAF‐2 displayed signs of yellowing around the upper region of the slip dress (Figure 3b). The CAF‐RCF blend exhibited the most significant discoloration, with browning and yellowing concentrated around fabric folds and scattered spotting across various parts of the textile (Figure 3c,d).

The primary method employed for initial fiber identification was Fourier transform infrared microscopy (*μ*‐FTIR) using the *μ*‐tip ATR attachment, which enabled precise analysis of both the textiles and individual fibers to detect the presence of textile blends. CAF displays distinct vibrational absorption bands at 1740 and 1360 cm^−1^, which are ascribed to the acetyl carbonyl stretching and the methyl deformation modes, respectively. In contrast, the spectra of RCF reveal a broad absorption feature ranging from 3750 to 3000 cm^−1^, indicative of hydroxyl (—OH) groups, alongside a prominent band at 1055 cm^−1^, which corresponds to C—O stretching in cellulose (for full band assignments please refer to supplemental information (SI2)). The analysis revealed that, except for the CAF‐RCF blend, all samples consisted of single‐fiber textiles. The CAF‐RCF blend was identified as a combination of cellulose acetate and regenerated cellulose woven together.

**Figure 4:**
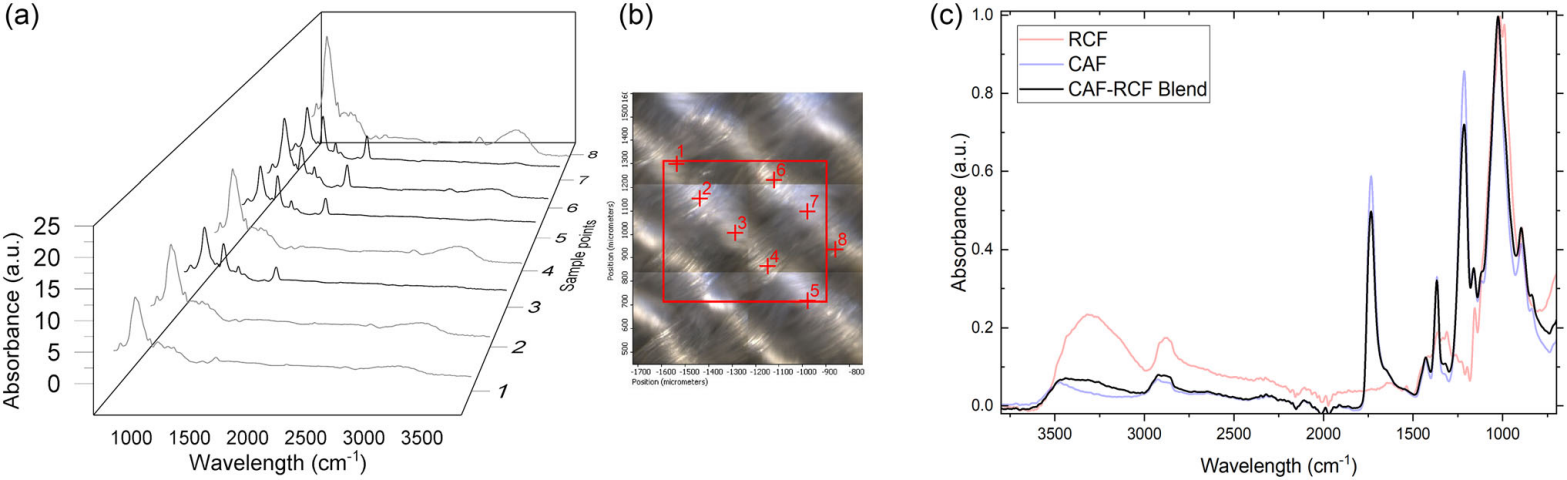
a) Infrared spectra for CAF‐RCF blend showing the alternating cellulose acetate and regenerated cellulose spectra based on measurement point in the textile blend. b) *μ*‐FTIR image showing measurement points in red. c) IR spectra of RCFs, CAFs, and CAF‐RCF blend comparing spectra using bench top ATR‐FTIR spectroscopy.

Identification of the textile blend in the CAF‐RCF sample was achieved through the selection and analysis of individual fibers using *μ*‐tip ATR‐FTIR (**Figure** **4**). When analyzed using standard benchtop ATR‐FTIR, the textile initially appeared to be composed entirely of cellulose acetate, highlighting the potential limitations in blend identification through conventional methods. Figure 4c compares the pure RCFs and CAFs spectra alongside the blended textile spectrum, illustrating the similarities in the spectral bands. The blend predominantly resembles cellulose acetate, with the exception of the hydroxyl peak at 3300 cm^−1^, which is broader in the CAF‐RCF spectrum.

### 2.2 Results of Artificial Ageing

#### 2.2.1 Mass Loss in CAFs, RCFs, and Blended Textiles

**Figure 5:**
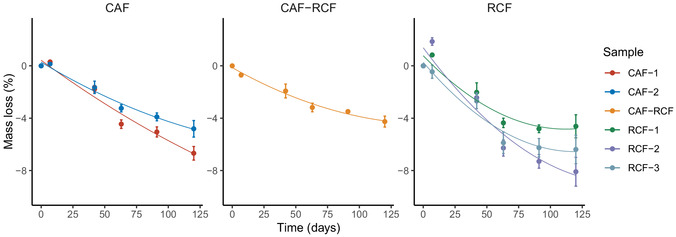
Mass loss (%) of CAF‐1 and CAF‐2 (left), CAF‐RCF (center), and RCFs‐1, RCF‐2, and RCF‐3 (right) samples over 120 days of ageing at 70 °C at 78% RH. Data are presented as the average of three sample repeats, with error bars indicating the standard deviation.

The mass change of the samples was monitored to quantify their physical loss of material, allowing for a comparative analysis of the weight loss among different fiber types over time. As shown in **Figure** **5**, all samples exhibited a decrease in mass over the 120‐day ageing period. On day 7, RCFs showed a slight mass increase, likely due to their moisture absorption, which we also suspect contributed to the higher variance in mass loss measurements. The RCF and CAF samples exhibited similar mass loss within their groups, while the CAF‐RCF blend showed significantly less final mass loss.

An analysis of variance (ANOVA) revealed significant differences in mass loss among fiber types (*p* = 0.001). Post hoc analysis using the Tukey honest significant difference (HSD) test revealed a significant difference in mass loss between the CAF and RCF‐CAF fibers (*p* = 0.006), with RCF‐CAF showing lower mass loss. No significant differences were observed between CAF and RCF (*p* = 0.162) or between RCF and CAF‐RCF (*p* = 0.105), though we may attribute the lack of statistical significance in the RCF samples to their high variance relative to the other fiber types.

As depicted in Figure 5, the mass loss of the RCF samples shows a relatively large range, with final mass losses of −4.62% for sample RCF‐1, −6.64% for the RCF‐3 and sample RCF‐2 showing the highest mass loss at −8.09%. Mass loss in the historical RCF samples appeared to plateau after ≈70 days of ageing, while the viscose reference (RCF‐3) continued to degrade at a steady rate. This discrepancy may be due to pre‐existing degradation in the historical textiles, leading to variations in mass loss trends. Both the CAF samples demonstrated comparable mass losses (*p* = 0.87), showing only slightly less mass loss than the RCF samples. These findings suggest that the degradation rate in terms of mass loss loosely follows the trend RCFs ≃ CAFs > CAF‐RCF blend.

Mass loss serves as a prominent indicator of material degradation, signifying the deterioration of constituents within a material over time. In the case of CAF, mass loss predominantly occurs through deacetylation, resulting in the release of volatile acetic acid.^[^ ^28^ ^]^ Conversely, RCF undergoes various degradation pathways. In RCF, the oxidation of hydroxyl groups can yield volatile degradation products such as aldehydes (e.g., furfural), ketones, and organic acids.^[^ ^29^, ^30^, ^31^ ^]^ Although these degradation products typically exhibit lower volatility compared to acetic acid, their accumulation, alongside the potential fragmentation of the cellulose backbone, may result in the generation of other low‐molecular‐weight volatile species, further contributing to mass loss.

Moreover, the intrinsic differences in material properties between CAF and RCF may influence their respective mass loss behaviors. The porous nature of the RCF matrix may promote the diffusion of volatile degradation products, even if these individual species possess lower volatility on a per‐molecule basis. Equally, in high moisture environments, such as those simulated under accelerated ageing conditions of 78% RH at 70 °C, moisture may enhance the transport of volatile degradation products from the material. This phenomenon may account for the slightly greater mass loss observed in RCF fibers relative to CAF.

#### 2.2.2 Color Change

**Figure 6:**
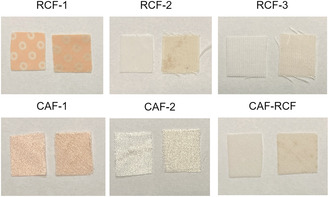
Before (left) and after (right) ageing of samples.

**Figure 7:**
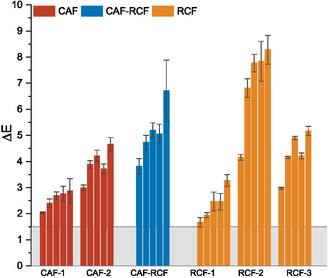
Δ*E*_2000_ for samples at day 7, 42, 63, 91, and 120 grouped by fiber type. The gray band represents the point at which color change is perceivable to the human eye (Δ*E*_2000_ above 1.5^[^ ^32^, ^34^ ^]^).

**Figure** **6** demonstrates the visual change in samples after ageing compared to an unaged control, highlighting the noticeable color difference due to ageing. Color change serves as a key indicator of material degradation, especially in heritage contexts where aesthetic shifts alter our perception of an object. Additionally, discoloration often occurs before mechanical breakdown, making it a valuable early warning sign of degradation.^[^ ^32^ ^]^ Color change in the samples was measured incrementally using the International Commission on Illumination (CIE) L*a*b* color space, a widely adopted metric for quantifying discoloration.^[^ ^32^, ^33^ ^]^ **Figure** **7** shows the Δ*E*_2000_ for each sample relative to the perceivable level (1.5) of color change, showing that after 7 days of ageing, all samples showed a notable color change. **Table** **2** presents the final Δ*E*_2000_ values after 120 days of accelerated ageing. All samples showed a Δ*E*_2000_ greater than 1.5 which is the limit for perceptible change.^[^ ^32^, ^34^ ^]^

**Table 2:** Table showing final Δ*E*_2000_ and their standard deviations (SD) for samples after 120 days of ageing.

Sample	Δ*E*_2000_	SD (*n* = 9)
RCF‐1	3.27	0.21
RCF‐2	8.28	0.55
RCF‐3	5.17	0.18
CAF‐1	2.88	0.44
CAF‐2	4.66	0.26
CAF‐RCF	6.71	1.17

An ANOVA revealed significant differences in Δ*E*_2000_ among different fiber types (*p* < 0.01). Post hoc analysis using Tukey's HSD test showed significant differences between CAF and RCF‐CAF (*p* < 0.00001) as well as between CAF and RCF (*p* < 0.00003), with both RCF‐CAF and RCF exhibiting greater color change than the CAF samples. However, no significant difference was observed between RCF and CAF‐RCF (*p* = 0.266).

The CAF and RCF samples revealed variability in discoloration between garments of the same fiber type. Notably, the garments exhibiting an initial pink hue demonstrated a substantially lower average color change than those with an initial white hue. RCF‐2 exhibited the most pronounced color change, reaching a final Δ*E*_2000_ of 8.28 this aligns with its higher mass loss. In contrast with the mass loss data, the CAF‐RCF blend showed the second‐highest color change at 6.71. This also suggests that blends may be more prone to discoloration than single‐fiber garments when compared with the CAF garments. This heightened susceptibility could result from interactions between the two fiber types during degradation.

#### 2.2.3 Detection of Deacetylation in Historic CAFs and CAF‐RCF

No spectral bands indicative of plasticizers were detected in the CAFs or CAF‐RCF blend samples. This aligns with previous reports, which indicate that CAFs do not contain plasticizers, as the fibers are produced by dissolving cellulose acetate in a solvent followed by direct extrusion, spinning, and drying processes.^[^ ^35^ ^]^ It has been established that plasticizer loss in cellulose acetate can increase its hydrophilicity, which accelerates deacetylation.^[^ ^16^ ^]^ The lack of plasticizers in these samples indicates that CAFs may be more susceptible to hydrolysis‐induced deacetylation than hard CA plastics with plasticizers, such as those used in social history objects or sculptures.

The DS for cellulose acetate was determined using ATR‐FTIR spectroscopy, based on the ratio of intensities of characteristic spectral bands, using the method developed by Da Ros et al. which utilizes the intensity ratio of the C—O—C asymmetric stretching in the ester group (located at 1215 cm^−1^) and the intensity of the C—O—C stretching in the pyranose ring (located at 1030 cm^−1^).^[^ ^36^ ^]^

Deacetylation occurs through a reaction between water and the acetyl group in CAFs, leading to the substitution of a hydroxyl group in place of the acetyl group, with acetic acid as a by‐product. This process results in a decrease in the DS of the cellulose acetate polymer, as illustrated in Equation (1)

(1) $$\text{ROAc}+H_{2}O\to \text{HOAc}+\text{ROH}$$

In this equation, OAc denotes the acetyl group (${\text{CH}}_{3}\text{COO}[chemistry single bond solid line]$), HOAc represents acetic acid, and ROH denotes the hydroxyl group attached to the polymer, with R representing the remainder of the polymer chain. As the DS decreases, an increase in hydroxyl content is expected. This was confirmed by monitoring the hydroxyl spectral band using the following equation

(2) $$\frac{\int {\text{3300 cm}}^{-1}}{\int {\text{2900 cm}}^{-1}}$$

where $\int {\text{3300 cm}}^{-1}$ is the integral of the hydroxyl band, and $\int {\text{2900 cm}}^{-1}$ is the integral of the C—H band which is used as an internal standard.

Deacetylation produces acetic acid which can further catalyze deacetylation, the acetic acid produced is also capable of degrading other materials.^[^ ^12^ ^]^ This may make other components of a garment such as buttons or zippers more vulnerable to degradation. Additionally, as deacetylation occurs the polymer becomes more susceptible to chain‐scission which results in a loss of mechanical integrity.^[^ ^37^ ^]^

**Figure 8:**
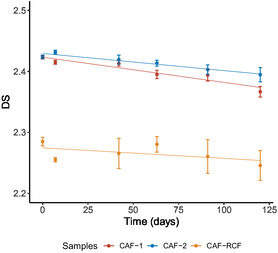
The change in the DS for CAF‐1 and CAF‐2 compared with CAF‐RCF over 120 days of ageing. Data are presented as the average of three sample repeats with three technical repeats on each sample, with error bars indicating the standard deviation.

**Figure** **8** shows the decline in DS over 120 days of ageing. The two CAF samples both began with an initial DS of 2.42. The existing literature states that the standard DS for CAFs is between 2.60 and 3.00.^[^ ^12^, ^38^ ^]^ The odor of acetic acid present on the garments before accelerated ageing suggests that some deacetylation has occurred during natural ageing, indicating that the DS was originally higher than 2.42. Over the course of 120 days, both samples exhibited a gradual reduction in DS, with CAF‐1 reaching a final DS of 2.36 and CAF‐2 a final DS of 2.39. Each condition showed a statistically significant reduction from their starting DS over the course of the 120 days of ageing (*p* ≤ 0.01, paired *t*‐test). The DS of the CAF‐RCF blend, determined using ATR‐FTIR spectroscopy due to its spectral similarity with pure CAFs, was initially lower at 2.28. It remains unclear whether this lower starting DS is inherent to the blend or a result of prior degradation. However, the DS of the CAF‐RCF blend showed minimal change over 120 days (*p* = 0.26), stabilizing at 2.25 after day 7 (For full spectra see Figure S3, Supporting Information).

**Figure 9:**
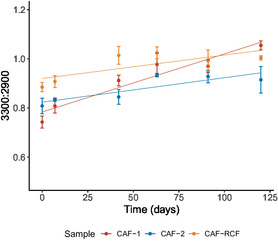
The change in the 3300:2900 ratio for CAF‐1 and CAF‐2 compared with CAF‐RCF over 120 days of ageing. Data are presented as the average of three sample repeats with three technical repeats on each sample, with error bars indicating the standard deviation.

In **Figure** **9**, the increase in the hydroxyl band area gives further evidence of the deacetylation process, as indicated by the DS trends. The final hydroxyl ratio for CAF‐1 was higher than for CAF‐2 (*p* ≤ 0.01), suggesting that the hydroxyl band may be more sensitive to deacetylation than DS measurements. This could provide an early indicator of degradation in CAF samples, while DS offers a more comprehensive assessment over time. However, the hydroxyl ratio exhibited greater variability than DS, likely due to residual moisture influencing the hydroxyl band. Similar to the DS results, the CAF‐RCF samples showed only a slight increase in the hydroxyl band, though it was not quite statistically significant (*p* = 0.083). The CAF‐RCF blend consistently displayed higher values compared to the pure CAF samples, likely reflecting the higher prevalence of RCF hydroxyl groups in the woven blend. The slight change in the CAF‐RCF hydroxyl ratio raises concerns about the effectiveness of current methods for detecting degradation in textile blends, particularly due to the observed visual degradation in the samples.

The findings indicate that ATR‐FTIR techniques serve as an effective methodology for monitoring degradation in single‐fiber garments, such as the CAF samples. However, this approach may prove insufficient for textiles comprising multiple fiber types, as evidenced by the CAF‐RCF blend analyzed in this study.

#### 2.2.4 Formation of Carbonyls in Historic RCF

The chemical degradation of the RCF samples was characterized by monitoring changes in the carbonyl band at 1730 cm^−1^. The extent of carbonyl formation was quantified using the following equation

(3) $$\frac{I_{{\text{1730 cm}}^{-1}}}{I_{{\text{2900 cm}}^{-1}}}$$

where $I_{{\text{1730 cm}}^{-1}}$ represents the intensity of the carbonyl band and $I_{{\text{2900 cm}}^{-1}}$ corresponds to the intensity of the C—H stretching band, used as an internal standard. The emergence of the carbonyl peak is associated with the formation of degradation byproducts, such as furfural, and the oxidation of the hydroxyl groups to aldehydes and ketones.

**Figure 10:**
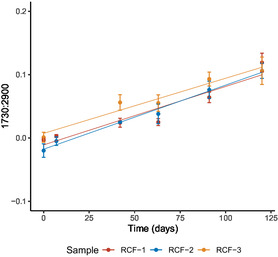
The change in the 1730:2900 ratio for RCF‐1, RCF‐2, and RCF‐3 (viscose reference) shows the increase in carbonyl peak over 120 days of ageing. Data are presented as the average of three sample repeats with three technical repeats on each sample, with error bars indicating the standard deviation.

**Figure** **10** illustrates the increase in carbonyl content as the RCFs samples underwent degradation over 120 days of accelerated ageing. This increase in the 1730:2900 ratio was the most significant change observed in the infrared spectra during this period. Notably, both naturally aged samples (RCF‐1 and RCF‐2), which had been subject to natural ageing for ≈70–80 years, exhibited a similar trend in carbonyl formation as RCF‐3, a newly prepared viscose reference sample. This suggests that the RCF samples had experienced minimal chemical degradation prior to the commencement of accelerated ageing. This observation is consistent with the mass loss data, which also indicated that all RCF samples followed a similar degradation rate (for full spectra see Figure S3, Supporting Information).

Research on native cellulose degradation, especially for paper degradation, shows that changes in the carbonyl band are reliable indicators of chemical degradation.^[^ ^39^, ^40^, ^41^ ^]^ This study suggests they are also effective markers for the degradation of RCFs. Given that the naturally aged RCF garments were in relatively good physical condition before accelerated ageing, these results may suggest that RCFs exhibit good chemical stability over long periods. This interpretation aligns with both the carbonyl data and the mass loss findings, which showed consistent degradation rates across both new and historic RCF samples.

#### 2.2.5 Comparative Surface Analysis of CAF‐RCF Blends in Aged and Unaged Conditions Using ***μ***‐FTIR

The bench‐top ATR‐FTIR analysis of the CAF‐RCF blend indicates minimal chemical changes, which contrasts with visual changes in the samples. To explore this discrepancy, further analysis was performed using *μ*‐FTIR in reflectance mode, allowing a comparison of the CAF component in the unaged control and after accelerated ageing. Reflectance mode offers the advantage of efficiently scanning more precise areas than ATR mode, thus providing enhanced accuracy in the surface chemistry on the individual fibers within the blend.

**Figure 11:**
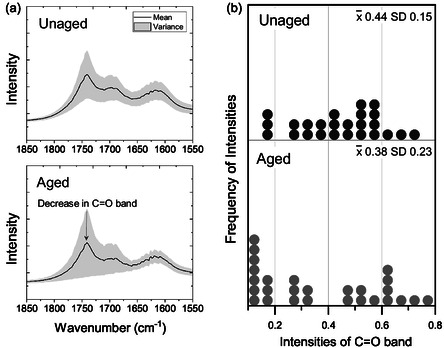
a) The carbonyl region with the mean spectra across all measurement positions with the variance in the band represented in gray, b) a dot plot showing the intensities for the carbonyl region, showing a higher frequency in low‐intensity bands for the aged samples. These results highlight a marked decrease in the C=O bond peak at 1740 cm^−1^ in aged samples.

Overall, both aged and unaged control samples display relatively similar spectra. However, **Figure** **11**a shows the variance in the carbonyl spectral band and reveals a marked decrease in the 1740 cm^−1^ band in the aged sample. The 1740 cm^−1^ band was chosen due to its clear resolution in the reflectance spectra, unlike the 1215 cm^−1^ C—O—C peak, which overlaps significantly with other peaks. A decrease in the acetyl carbonyl indicates deacetylation has occurred.^[^ ^24^ ^]^ This reduction is further supported by Figure 11b which shows a higher frequency of lower intensity peaks after ageing, suggesting a heterogeneous decrease of the C=O bond at 1740 cm^−1^ across the sample surface. Such a decrease aligns with a reduction in the carbonyl group, suggesting that deacetylation in the CAFs component may be more pronounced than previously indicated by bench‐top ATR‐FTIR measurements. These results highlight the need for specific methods to monitor the degradation of blends within collections and illustrate potential pitfalls that could lead to the misidentification of a garment's chemical condition.

#### 2.2.6 Microscopy Analysis of Fiber Morphology

**Figure 12:**
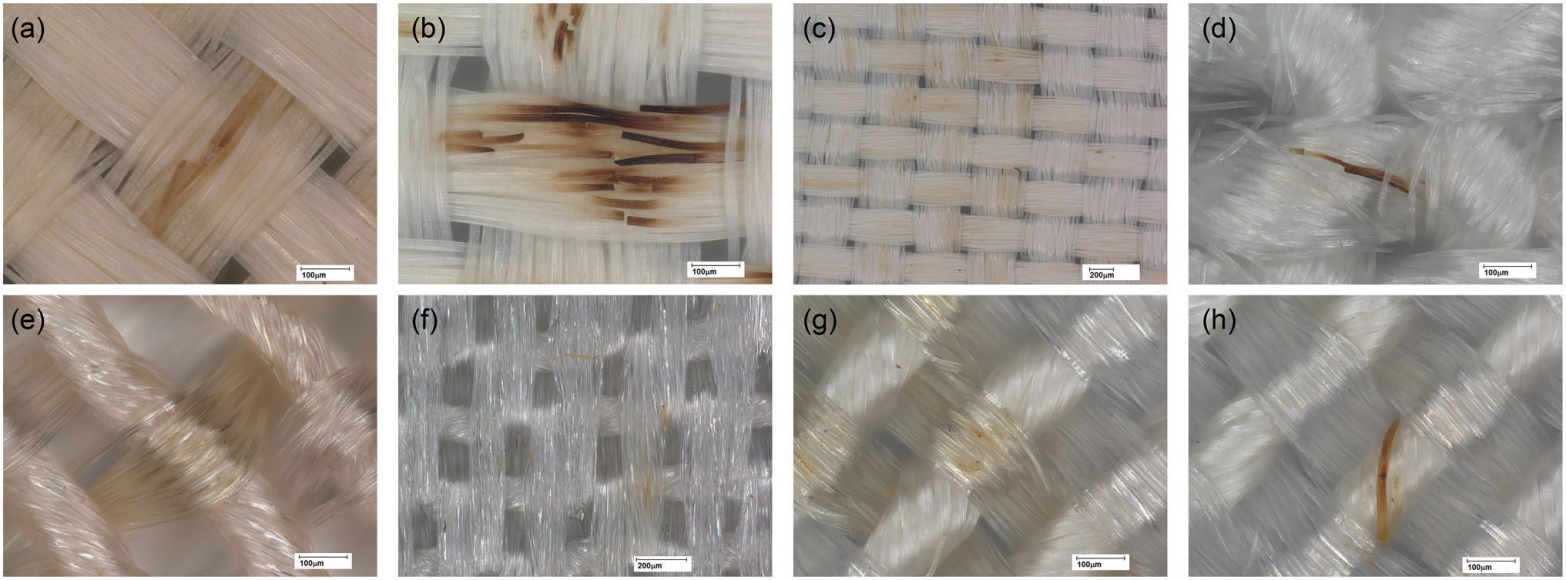
Images of signs of degradation on RCFs after ageing: a) RCFs‐1, b,c) RCF‐2 and d) RCF‐3, e) CAF‐1 showing signs of yellowing, f) CAF‐2 showing signs of yellowing and g) CAF‐RCF showing yellowing on the CAFs component, and h) CAF‐RCF showing browning of the RCFs component.

Microscopy was used to monitor potential changes in fiber morphology during the ageing process. **Figure** **12** illustrates details of physical degradation in each RCF sample, with the most prominent changes observed as darkening along the fiber shafts. In many cases, this darkening was followed by fiber breakage. Notably, the RCF‐2 samples exhibited significantly more instances of breakage and darkening compared to the other RCF samples (RCF‐1 and RCF‐3). A progression in degradation could be observed, with initial yellowing and discoloration of fiber sections (Figure 12c) followed by subsequent darkening and fracturing of the fibers (Figure 12b). These changes were often localized, spreading from a specific section, although no consistent pattern in the spread was discernible. Interestingly, RCF‐3, the newly prepared reference sample, exhibited significantly fewer visible signs of degradation compared to RCFs‐1 and RCF‐2, despite undergoing similar levels of mass loss and chemical changes.

Figure 12 presents evidence of physical degradation in the CAFs and CAF‐RCF blend samples. Unlike the RCF fibers, both CAF samples exhibited almost no observable physical changes (Figure 12e,f) when compared to their unaged control samples. The only visible changes were minimal yellowing, and the fibers otherwise appeared almost pristine. These findings contrast with the chemical data, which indicated a significant reduction in the DS after ageing. This discrepancy raises questions about the reliability of physical observation methods alone in detecting early stages of degradation in CAF garments.

In contrast, the CAF‐RCF blend exhibited some of the most pronounced physical changes (Figure 12g,h). The fibers with higher luster were identified as CAFs, while the smoother, more opaque fibers were identified as RCFs. The CAFs in the blend displayed more frequent yellowing than the single‐fiber CAF samples, although this yellowing did not correlate with fiber fractures, as seen in the RCF samples. This suggests that the blended fabric may be more prone to yellowing than the single‐fiber CAF garments. The RCF component of the blend exhibited signs of degradation comparable to those of the other RCF samples, including discoloration and occasional fractures. However, in general, the CAF fibers in the blend showed more signs of degradation than the RCF fibers.

## 3 Discussion

This study addresses the emerging issue of understanding the degradation processes of MMCF in historical garments, particularly focusing on CAFs, RCFs, and their blends (CAF‐RCF). Given the increasing prevalence of these materials in museum collections, understanding their degradation mechanisms is important for developing effective conservation strategies and long‐term storage. This research utilizes naturally aged garments deaccessioned from museums as case studies to provide realistic insights into how their degradation progresses within the garment's lifetime. Accelerated ageing is used to evaluate their degradation markers and explore existing monitoring techniques. The study contributes to a more comprehensive understanding of the complexities of MMCF degradation and the associated challenges in the conservation and preservation of these materials.

The findings reveal notable differences in the degradation patterns of CAFs, RCFs, and their blends. Mass loss data indicated that RCFs degrade similarly to CAFs, with the blends exhibiting lower degradation rates. Notably, the absence of plasticizers in CAFs may have rendered them more susceptible to hydrolytic deacetylation than hard plastics, as evidenced by a marked decrease in the DS. Despite these chemical changes, the physical degradation of CAFs, such as darkening or fiber breakage, was minimal compared to RCFs. This highlights a possible challenge in detecting early‐stage degradation in CAF garments through physical observation alone, underscoring the need for chemical monitoring in combination with condition reports. Moreover, color change analysis demonstrated that RCF fibers are more prone to discoloration than other MMCFs. The CAF‐RCF blends exhibited pronounced yellowing, potentially due to interactions between the two fiber types. These findings suggest that fiber blends may pose unique degradation challenges, necessitating tailored monitoring techniques to understand their long‐term stability.

The decrease in DS in CAFs, while a clear marker of chemical degradation, is not fully mirrored by physical changes as discoloration was often less significant than in the RCFs and blend. This raises concerns about the reliance on physical markers in assessing the condition of CAF garments, as objects may appear visually stable while experiencing marked chemical degradation. This indicates that visible changes may not reliably indicate chemical degradation in CAF garments. Current tools, such as condition reports, may not adequately capture this latent degradation highlighting the need for a multianalytical approach to assess garment condition. Future research should focus on establishing the relationship between DS reduction and mechanical integrity, as understanding how chemical and physical changes interact will further elucidate the stability of CAFs.

The CAF‐RCF blends yield the second‐highest ΔE of 6.71, yet chemical analysis via IR spectroscopy detected minimal spectral changes. This suggests that conventional IR‐based monitoring methods for blends may not be sufficiently sensitive to detect early‐stage chemical changes in fiber blends. The use of techniques such as *μ*‐FTIR may offer a more precise solution to the chemical monitoring of fiber blends. The findings further indicate that fiber blends may degrade more rapidly than individual fibers, as the blend showed an increased yellowing of CAFs in the blend compared to single‐fiber CAF samples. This may result from interactions between the constituent fiber types. This presents a significant challenge, as existing techniques designed for single‐fiber textiles may not adequately address the complexities of blended materials. The study calls for the development of more sensitive and fiber‐specific monitoring techniques to better understand the degradation dynamics in such materials.

Future research should aim to expand the scope of these investigations by incorporating a broader range of MMCF textiles, including those with varying pigment compositions, fiber treatments, and weave structures, to develop a more holistic understanding of their degradation behavior. Additionally, exploring a wider range of ageing conditions, including variations in temperature, humidity, and light exposure, would provide insights into the environmental interactions that influence the degradation of MMCF textiles. This expanded approach would support the development of more effective monitoring techniques tailored to the specific needs of MMCF‐based heritage objects.

## 4 Experimental Section

4.1

4.1.1

##### Samples

Samples were taken from a selection of five historical garments all dating between 1940 and 1950 which had been deaccessioned from the London Museum. Two of the five garments were cellulose acetate (CAF‐1, CAF‐2), two of the garments were viscose rayon (RCF‐1, RCF‐2), and one garment was viscose rayon and cellulose acetate blended together (CAF‐RCF). A reference sample set of new viscose samples (RCF‐3) was included for comparison with historical RCFs, we were unable to obtain reference samples for the other fiber types. All textiles were characterized fully before ageing. The fabric was cut into swatches of 1.5 cm^2^ with a thickness of ≈0.05 cm. Each garment, including the reference sample (RCF‐3), had three repeat samples prepared and aged, three repeats of each sample were also kept at 5 °C as controls for comparison.

##### Accelerated Ageing Method

To degrade the samples, they were aged at 78% relative humidity (RH) and 70 °C for 120 days. To achieve the desired RH, a saturated salt solution of potassium chloride (KCl) was used. Samples were suspended in a closed container with the salt solution and aged in an oven set to 70 °C, see Figure S5, Supporting Information for a diagram of the ageing container. Samples were removed from the oven at intervals analyzed, then placed back in the oven for ageing. Mass loss was measured after samples had cooled to room temperature (20 °C) after 30 min out of the oven.

##### ATR‐FTIR

Due to the absorbent nature of textiles, they retain a high level of residual moisture, which is further amplified when exposed to high‐humidity environments. Moisture trapped in the textiles has been found to impact IR measurements, changing the intensity of spectral bands. Consequently, samples were oven‐dried at their ageing temperature for 60 min before measurements were taken to minimize moisture spectral interference with the measurements. Infrared spectra were collected of all the samples before and after ageing in the frequency range of 4000 and 400 cm^−1^ using a Bruker Alpha Platinum‐ATR‐FTIR spectrometer equipped with a diamond crystal cell. Spectra were measured using 32 scans with a spectral resolution of 4 cm^−1^. Three repeat measurements were taken per sample and then averaged with all sample repeats totaling nine measurements per average for each sample type, each spectrum was baseline corrected and normalized. Spectra were baseline corrected by setting the average absorbance from 2000 to 2200 cm^−1^ to zero. Spectra were normalized to the maximum peak, which is the C—O band at 1055 cm^−1^ for both RCF, CAF, and blend samples. Spectra were averaged, baseline corrected, and normalized using MATLAB2023a.

##### Color Measurements

Color measurements were taken at intervals using a spectrophotometer (X‐Rite Ci6XS Handheld Spectrophotometer). The CIELab color space defined by the CIE was used to monitor color change.^[^ ^42^ ^]^ The CIELab color space is expressed using L* to indicate black (0) to white (100), a* to indicate green (−) to red (+), and b* to indicate blue (−) to yellow (+) color change. Three repeat measurements were taken on each sample type which each had three repeats, and an average and standard deviation was calculated (*n* = 9). The change in color of the samples, Δ*E*_2000_ was also calculated between the initial value measured before and after the ageing periods.^[^ ^42^ ^]^ Δ*E*_2000_ was calculated in the Color iQC 10 Software.

##### Digital Microscopy

Changes in fiber morphology were monitored using a Keyance VHX7000 digital microscope in transmitted light mode. Microscopy was used to monitor the degradation of individual fibers through changes in fiber morphology and the formation of deposits on the surface.

##### *μ*‐FTIR

*μ*‐FTIR was used to initially identify the textiles using *μ*‐tip ATR mode. A Thermo Scientific Nicolet iN10 MX was used with a liquid nitrogen‐cooled mercury cadmium telluride (MCT) detector for surface measurements using 64 scans and 4 cm^−1^ resolution. The aperture was set to 200 × 200 μm^2^ with points selected manually for measurements. The spectral intensity was normalized using OMNIC Picta software and processed in OriginPro 2024. Discreet spectra analysis was used to establish the composition of the garments, determining whether they consisted of single or blended fiber types. This identification was achieved through the interpretation of spectral band assignments, see Figure S2, Supporting Information for full band assignments.

For the mapping of chemical changes in the CAF‐RCF blend, reflectance mode was utilized in combination with line mapping using 124 scans and 4 cm^−1^ resolution. An aperture of 200 × 200 μm^2^ was used with a step size of 200 μm. The spectra were processed using the Kubelka–Munk (KM) transform.^[^ ^43^ ^]^ The spectral intensity was normalized and processed in R to create contour maps to study individual regions of the spectrum. Spectra were normalized to the maximum peak, which is the C—O band at 1055 cm^−1^ for both RCF, CAF, and blend samples.

##### Statistical Methods

To analyze the effect of fiber type and time on mass loss and color change, a two‐way ANOVA was conducted with a CI of 95%. Observations were weighted by the inverse of the squared standard deviation to account for variability in measurement precision. This approach ensures that measurements with smaller standard deviations (higher precision) were given more weight in the analysis, while those with larger standard deviations contributed less. To further explore differences among fibers, Tukey's HSD test was performed, this post hoc test identifies pairwise differences while controlling for family‐wise error rate. The results of the Tukey HSD test were inspected to determine statistically significant differences between fiber.

To establish the statistical significance of the results, we employed a combination of pair *t*‐tests and two‐sample *t*‐tests with a confidence level (CI) of 95% for the chemical data. This approach allowed us to effectively compare the results and identify any significant differences between them.

## Conflict of Interest

The authors declare no conflict of interest.

## Supporting information

Supplementary Material

## Data Availability

The data that support the findings of this study are available from the corresponding author upon reasonable request.
