# Supplementary material for: Investigating the Degradation of Historical Man‐Made Cellulose‐Derived Textiles via Accelerated Ageing
Source: Chempluschem. 2025 May 7;90(6):e202500025. doi: 10.1002/cplu.202500025 (PMC12143456; doi:10.1002/cplu.202500025)
Supplement: Supplementary file 1 — Supplementary Material [file CPLU-90-e202500025-s001.pdf]

Supporting Information

SI1: Garment Images and Descriptions

RCF-1

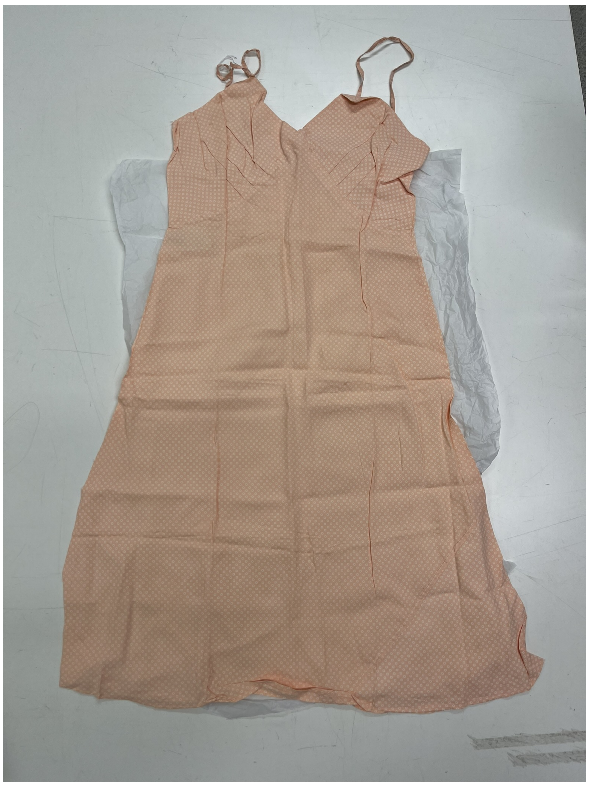

Figure S1: Slip dress component of RCF-1.

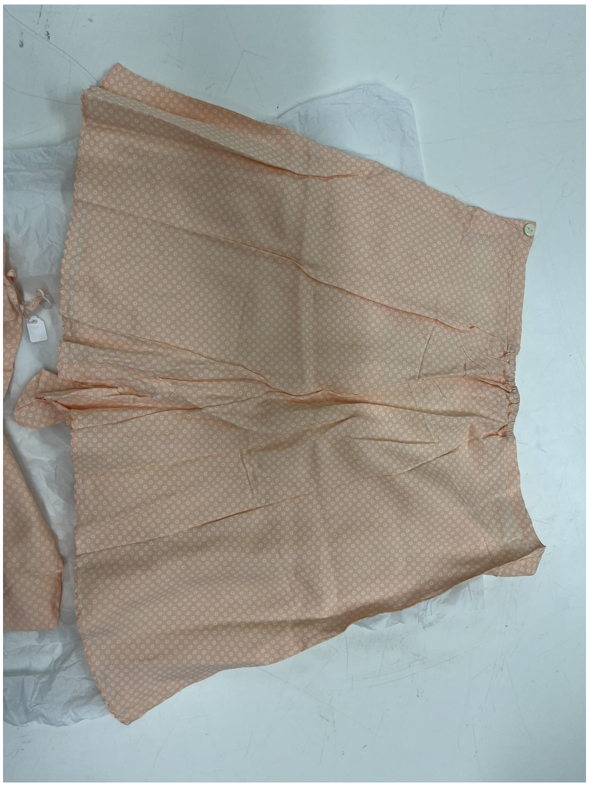

Figure S2: French knickers component of RCF-1.

RCF-2

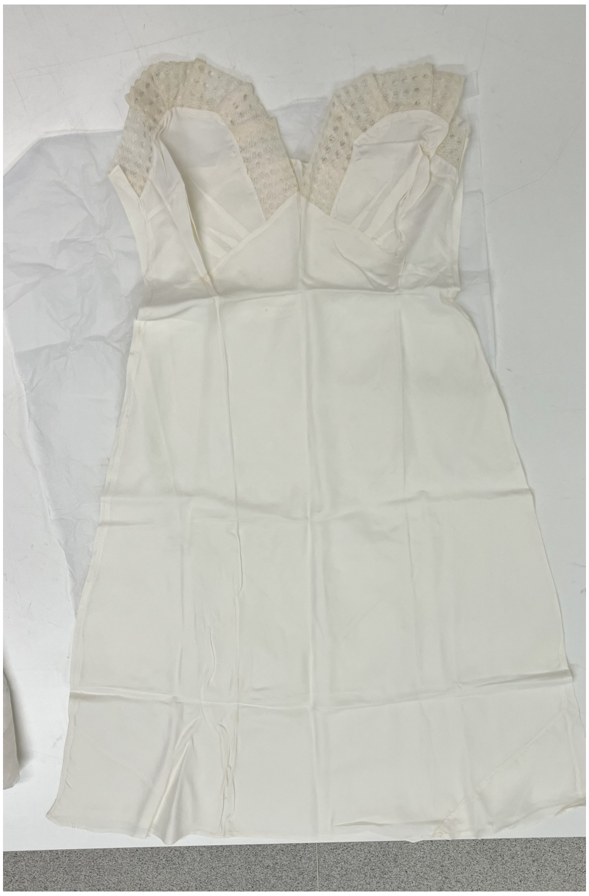

Figure S3: Slip component of RCF-2.

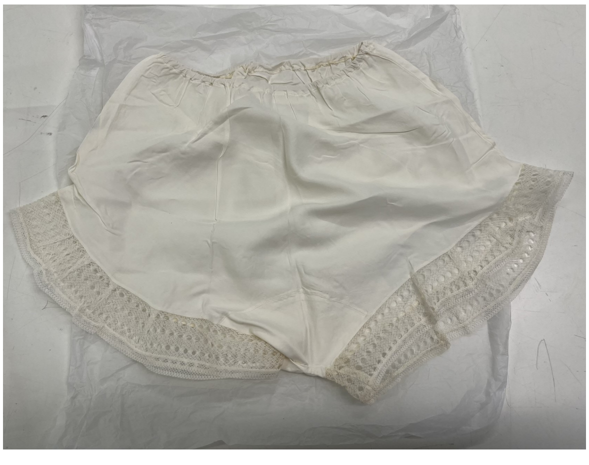

Figure S4: French knicker component of RCF-2.

CAF-1

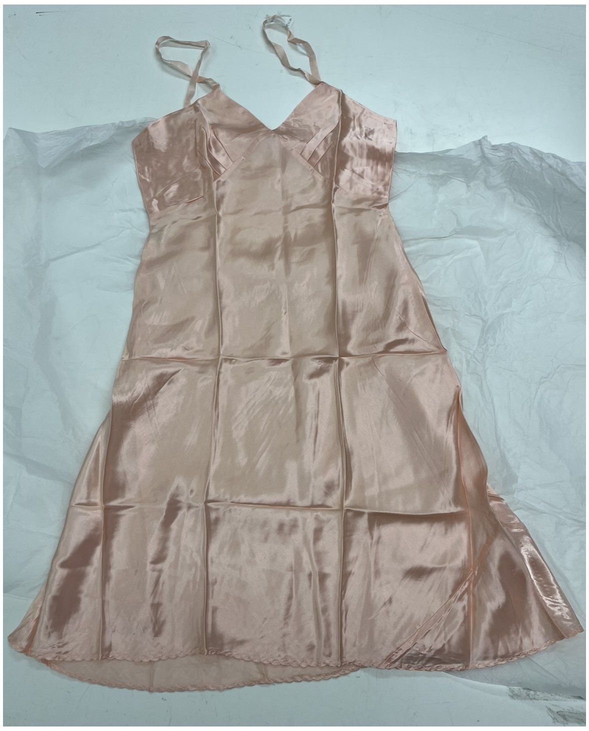

Figure S5: Slip component of CAF-1.

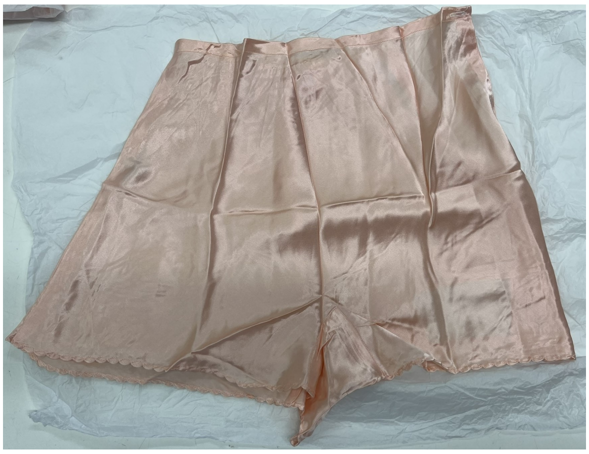

Figure S6: French knicker component of CAF-1.

CAF-2

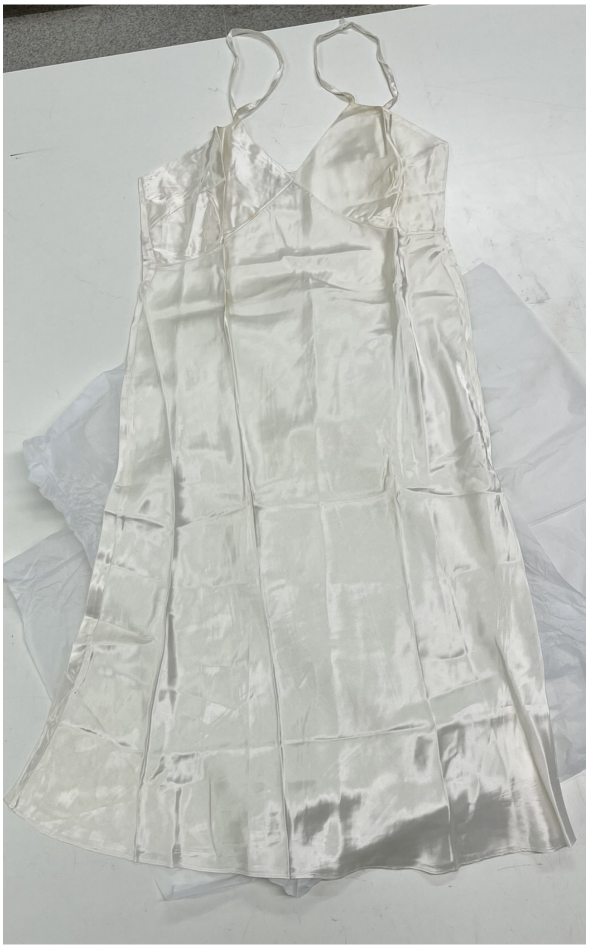

Figure S7: Slip component of CAF-2.

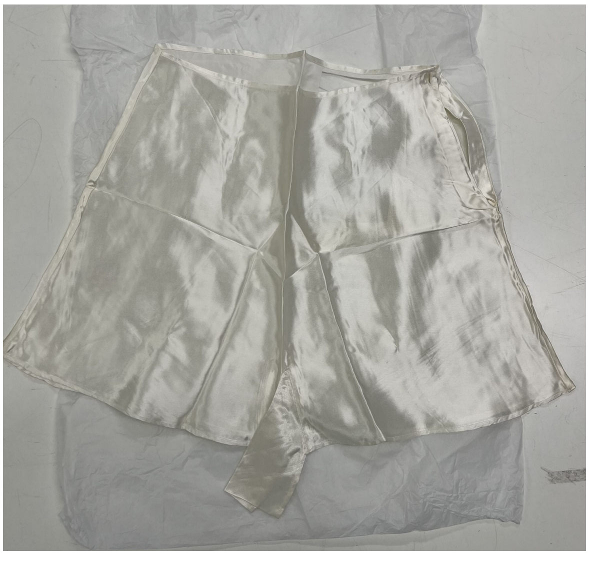

Figure S8: French knicker component of CAF-2.

CAF-RCF

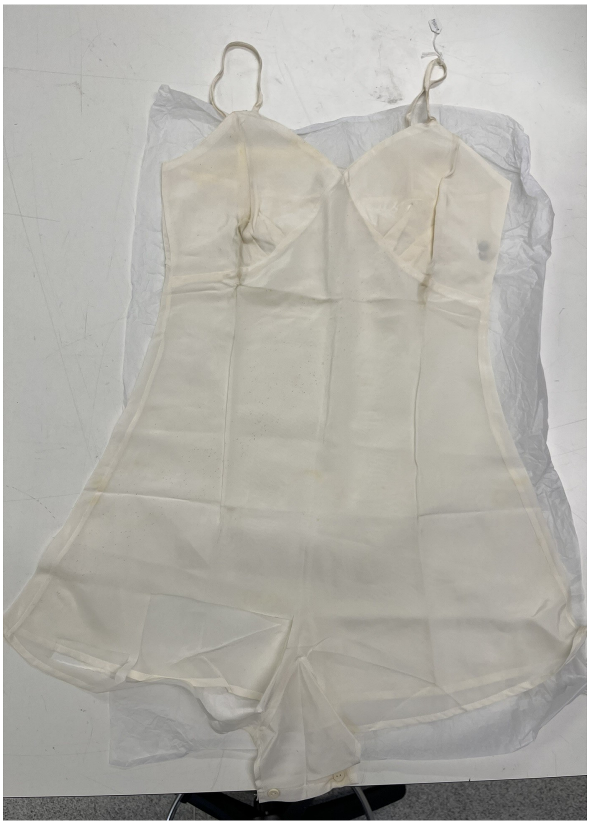

Figure S9: Slip of CAF-RCF.

SI2: Absorption Band Assignment of CAF and RCF Fibre Types

Table S1: Table of main absorption bands for CAF and RCF fiber types.[2, 4, 1, 5, 3]

Wavenumber (cm^{-1})	Assignment	Fibre Type
3300 (broad)	ν -OH intramolecular hydrogen bonds	CAF, RCF
3000-2850	ν C-H	RCF, CAF
1740	ν C=O acetyl group	CAF
1360	δ C-H in CH_3 in the acetyl group	CAF
1111	ν_{as} glucose ring	RCF
1055	ν C-O	RCF
1035	ν C-O	RCF, CAF
892	ν C_1-O-C_4 glycosidic link	RCF

SI3: Full Infrared Spectra at Intervals

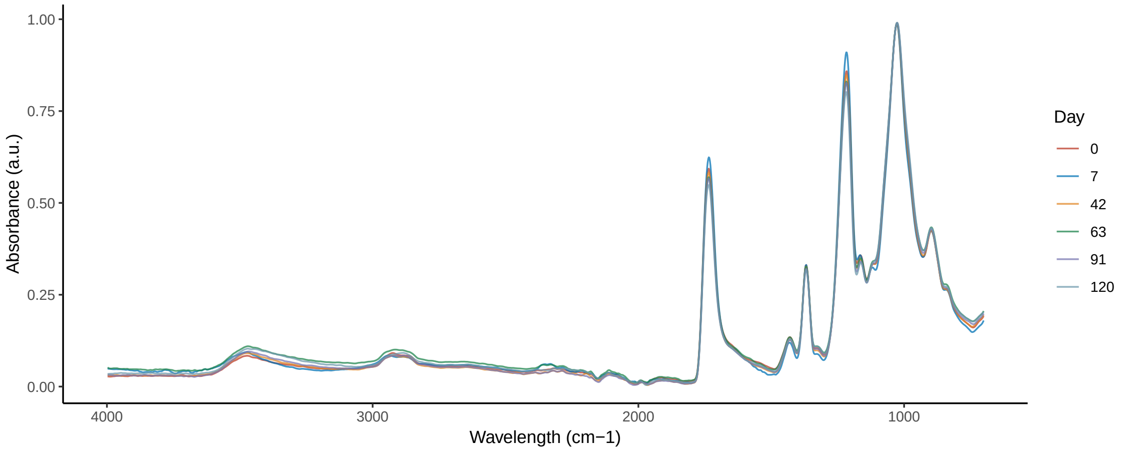

Figure S10: Infrared spectra for CAF-1 samples were obtained at 0, 7, 42, 63, 91, and 120, presenting the average spectrum from repeated measurements.

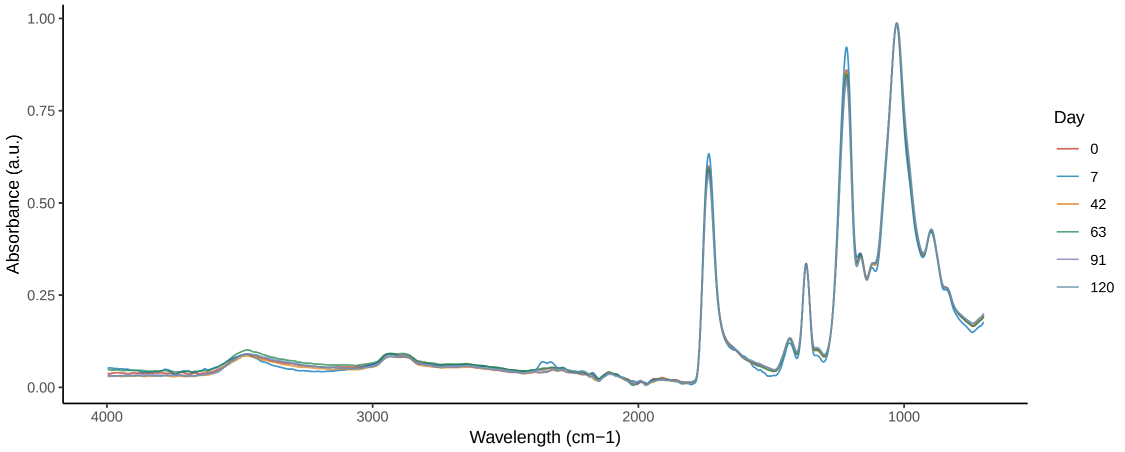

Figure S11: Infrared spectra for CAF-2 samples were obtained at 0, 7, 42, 63, 91, and 120, presenting the average spectrum from repeated measurements.

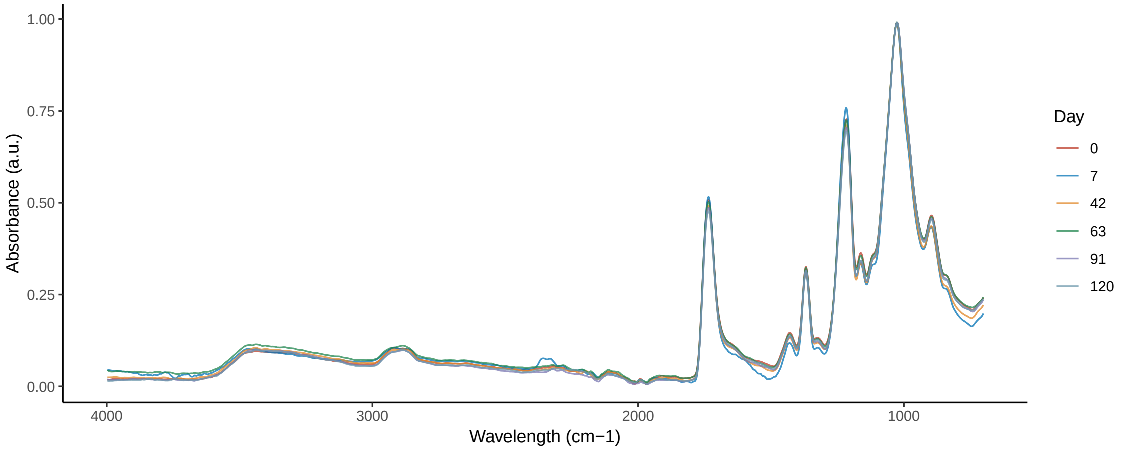

Figure S12: Infrared spectra for CAF-RCF samples were obtained at 0, 7, 42, 63, 91, and 120, presenting the average spectrum from repeated measurements.

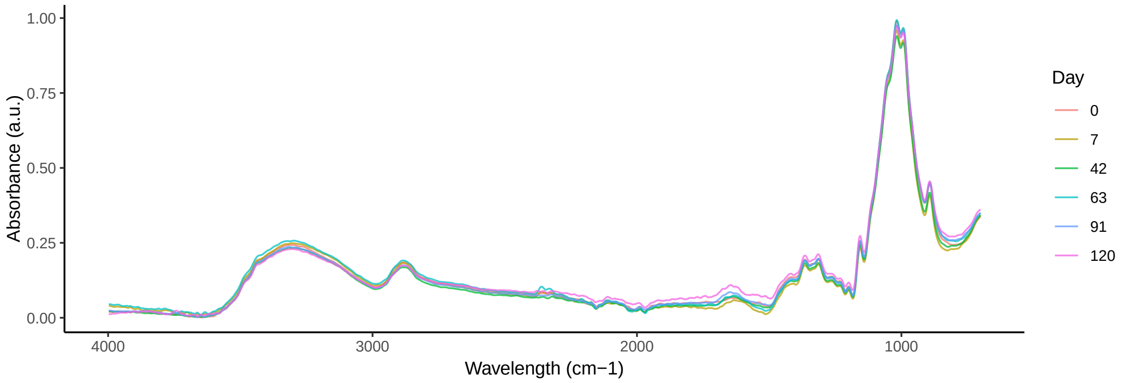

Figure S13: Infrared spectra for RCF-1 samples were obtained at 0, 7, 42, 63, 91, and 120, presenting the average spectrum from repeated measurements.

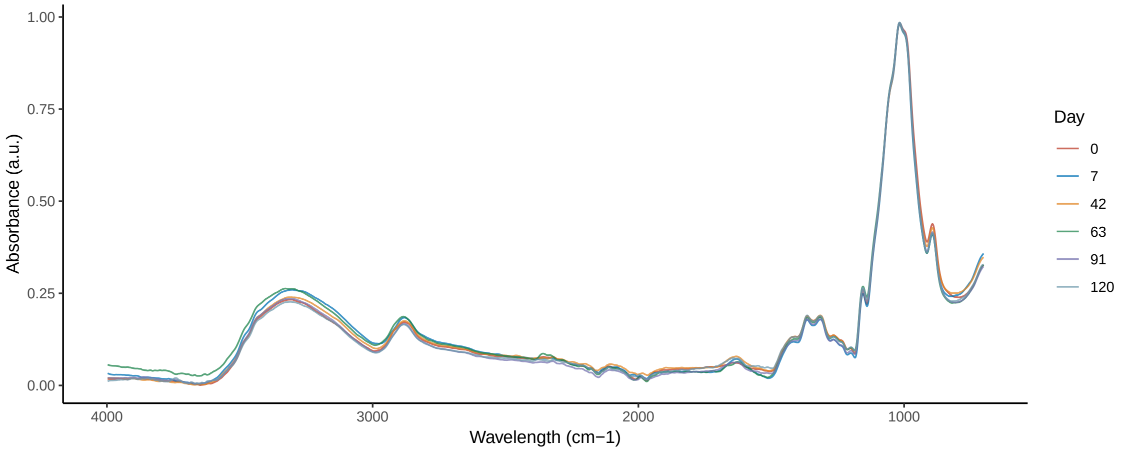

Figure S14: Infrared spectra for RCF-2 samples were obtained at 0, 7, 42, 63, 91, and 120, presenting the average spectrum from repeated measurements.

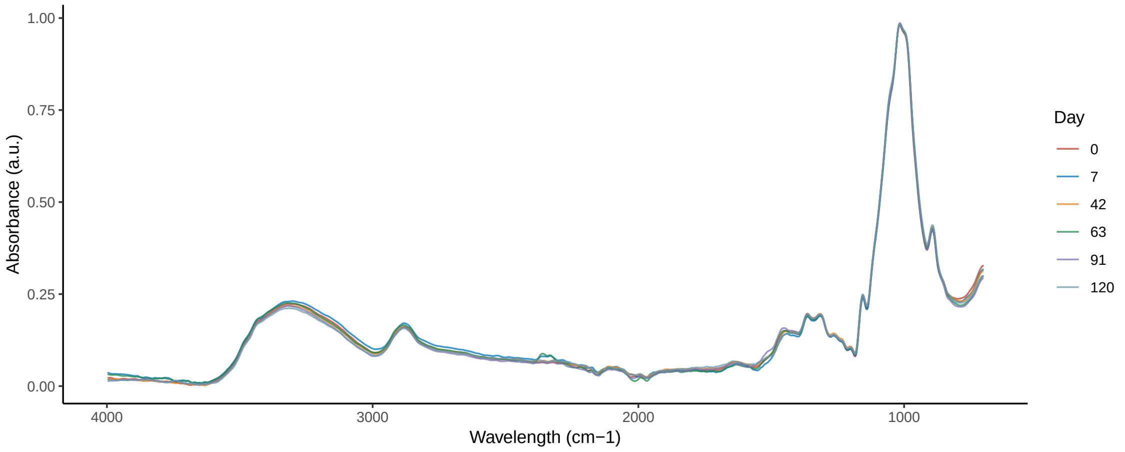

Figure S15: Infrared spectra for RCF-3 samples were obtained at 0, 7, 42, 63, 91, and 120, presenting the average spectrum from repeated measurements.

SI4: L*a*b* Color Analysis

The directional changes in colour are presented in Figures S16 and S17. Figure S16 illustrates the variation in L* (lightness, where a decrease signifies darkening) over 120 days of ageing. The data indicate a general reduction in L*, suggesting progressive darkening across the samples. Notably, no significant changes were observed in a* values for any of the samples, indicating colour stability in the red-green axis.

The most pronounced observation is the rapid darkening exhibited by RCF-2, which suggests that it may be more susceptible to discolouration relative to both RCFs-1 and RCF-3. Interestingly, despite being a new reference sample, RCF-3 exhibited a slower darkening rate than the other RCF samples, indicating potentially better stability or reduced degradation under these conditions. The CAF-RCF blend displayed an intermediate rate of L* decline, implying that the combination of CAFs and RCFs may result in mutual degradation effects, possibly due to interactions between the two fibre types that influence the extent of discolouration.

Figure S17 highlights an increase in b* values, indicating yellowing across all samples. The similarity in the rate of change for L* and b* values suggests that yellowing occurs concurrently with darkening. This trend was consistent across all samples except for RCF-1, which showed negligible change in b* values. This stability in RCF-1's b* may be attributed to its initial hue rather than a reduced susceptibility to degradation.

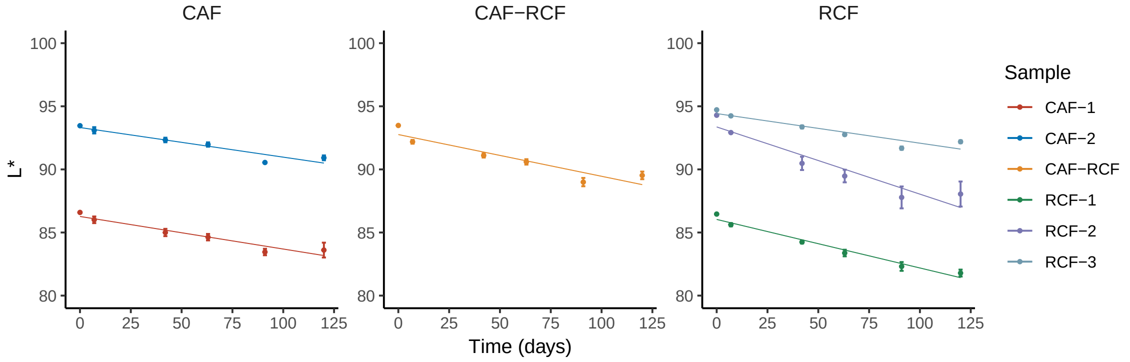

Figure S16: Plot showing the change in L* (light $\rightarrow$ dark) for samples over 120 days of ageing.

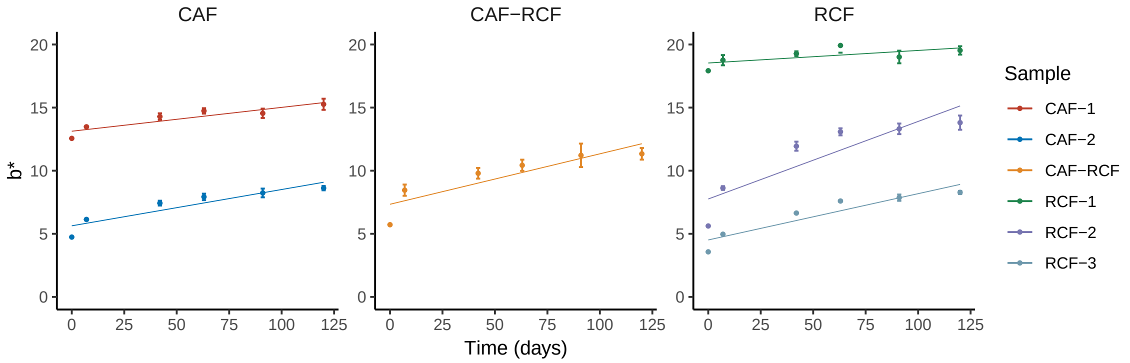

Figure S17: Plot showing the change in b* (blue $\rightarrow$ yellow) for samples over 120 days of ageing.

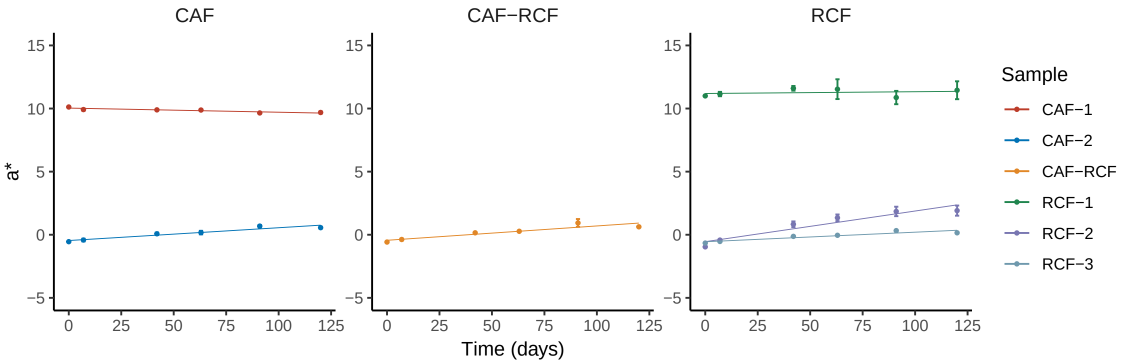

Figure S18: Plot showing the change in a* (red → green) for samples over 120 days of ageing.

SI5: Scheme for Sample Ageing Set-Up

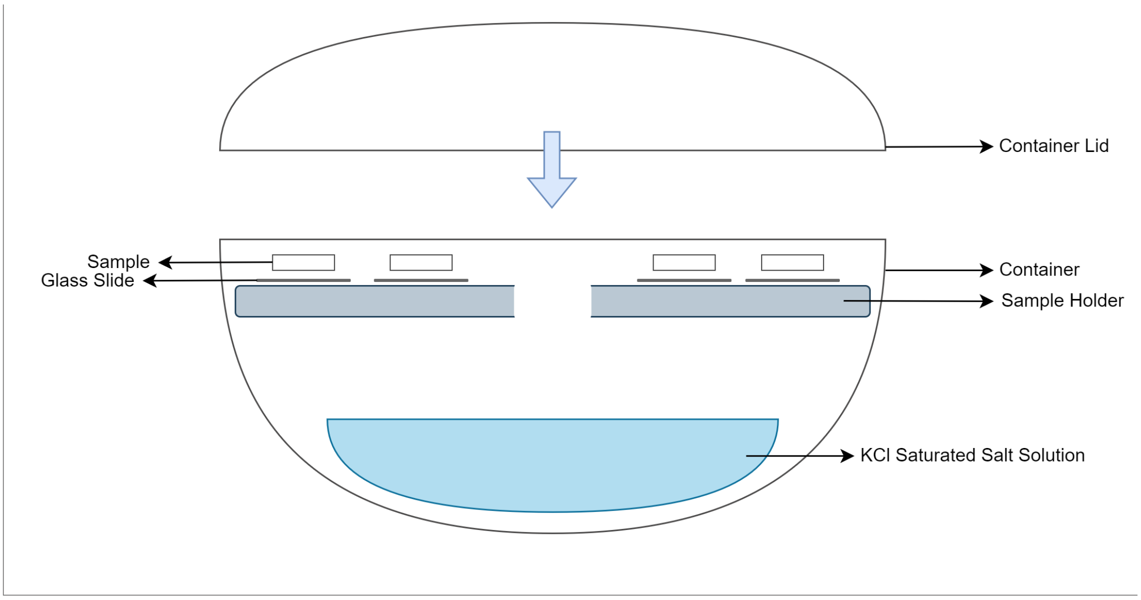

Figure S19: Diagram illustrating the ageing setup for a container with a salt solution to age samples in the oven.

References

[1] Abdellatif Boukir, Somia Fellak, and Pierre Doumenq. "Structural characterization of Argania spinosa Moroccan wooden artifacts during natural degradation progress using infrared spectroscopy (ATR-FTIR) and X-Ray diffraction (XRD)". In: *Heliyon* 5.9 (Sept. 2019). ISSN: 24058440. DOI: 10.1016/J.HELIYON.2019.E02477.

[2] F. Carrillo et al. "Structural FTIR analysis and thermal characterisation of lyocell and viscose-type fibres". In: *European Polymer Journal* 40.9 (Sept. 2004), pp. 2229–2234. ISSN: 0014-3057. DOI: 10.1016/J.EURPOLYMJ.2004.05.003.

[3] Pengfei Fei et al. "Quantitative analysis of cellulose acetate with a high degree of substitution by FTIR and its application". In: *Analytical Methods* 9.43 (Nov. 2017), pp. 6194–6201. DOI: 10.1039/c7ay02165h.

[4] D. Ołdak et al. "Photo- and Bio-Degradation Processes in Polyethylene, Cellulose and their Blends Studied by ATR-FTIR and Raman Spectroscopies". In: *Journal of Materials Science 2005 40:16* 40.16 (2005), pp. 4189–4198. ISSN: 1573-4803. DOI: 10.1007/S10853-005-2821-Y. URL: https://link.springer.com/article/10.1007/s10853-005-2821-y.

[5] Diana C. Rambaldi et al. "Effect of thermal and photochemical degradation strategies on the deterioration of cellulose diacetate". In: *Polymer Degradation and Stability* 107 (Sept. 2014), pp. 237–245. ISSN: 0141-3910. DOI: 10.1016/J.POLYMDEGRADSTAB.2013.12.004.
